# BDNF Val^66^Met Polymorphism Is Associated with Self-Reported Empathy

**DOI:** 10.1371/journal.pone.0149911

**Published:** 2016-02-22

**Authors:** Vincent Taschereau-Dumouchel, Sébastien Hétu, Anaït Bagramian, Alexandre Labrecque, Marion Racine, Yvon C. Chagnon, Philip L. Jackson

**Affiliations:** 1 École de psychologie, Université Laval, Québec, Québec, Canada; 2 Centre interdisciplinaire de recherche en réadaptation et intégration sociale (CIRRIS), Québec, Québec, Canada; 3 Centre de recherche de l’institut universitaire en santé mentale de Québec (CRIUSMQ), Québec, Québec, Canada; 4 Human Neuroimaging Laboratory, Virginia Tech Carilion Research Institute, Roanoke, Virginia, United States of America; 5 Département de Psychiatrie et des Neurosciences, Université Laval, Québec, Québec, Canada; University of Wurzburg, GERMANY

## Abstract

Empathy is an important driver of human social behaviors and presents genetic roots that have been studied in neuroimaging using the intermediate phenotype approach. Notably, the Val66Met polymorphism of the Brain-derived neurotrophic factor (*BDNF*) gene has been identified as a potential target in neuroimaging studies based on its influence on emotion perception and social cognition, but its impact on self-reported empathy has never been documented. Using a neurogenetic approach, we investigated the association between the *BDNF* Val66Met polymorphism and self-reported empathy (Davis’ Interpersonal Reactivity Index; IRI) in a sample of 110 young adults. Our results indicate that the *BDNF* genotype is significantly associated with the linear combination of the four facets of the IRI, one of the most widely used self-reported empathy questionnaire. Crucially, the effect of *BDNF* Val66Met goes beyond the variance explained by two polymorphisms of the oxytocin transporter gene previously associated with empathy and its neural underpinnings (*OXTR* rs53576 and rs2254298). These results represent the first evidence suggesting a link between the *BDNF* gene and self-reported empathy and warrant further studies of this polymorphism due to its potential clinical significance.

## Introduction

Social interactions are tightly linked to survival, so much so that the complexity of social environments has been proposed as one of the main factors driving the increasing size of primates’ brains across evolution [1]. Accordingly, an impressive amount of data indicates specific neural processes for social perception and social cognition that are highly conserved across species [2]. Empathy, defined as the capacity to understand and share the actions, intentions and emotions of others [3, 4], is one of these processes that has been shown to be deeply rooted in the neurobiology [5] and the evolution of the human brain [2]. For example, a twin study found that in 24 and 36 month-old infants, genetic factors accounted respectively for 34% and 47% of the variance in empathy [6]. However, current evidence from the scientific literature does not fully detail the specific genetic variants contributing to the observed interindividual variability in empathy.

Empathy is conceptualized as a multilayered construct where higher order social cognitive processes rely partly on social perception to allow the understanding of others (see [7]). Accordingly, empathy often begins with the perception of social stimuli that can trigger perception-action coupling (Preston & De Waal, [8]; also referred to as shared representations: Decety and Jackson [3]) and even emotional contagion, a process thought to be evolutionarily old as it has been observed in non-primate species such as mice [9]. In humans, a wide network of structures that includes the amygdala nuclei has been shown to be crucial for the perception of socially and emotionally salient stimuli (see [5, 10]). This network feeds sensory information to cortical structures that have been shown to support the processing of higher order empathic processes in the brain (see [3, 5]). These processes are typically divided in two broad categories: affective and cognitive components of empathy. On the one hand, the affective component of empathy allows individuals to automatically form shared representations of the feelings of others while preserving the self-other distinction, which differentiate this component of empathy from emotional contagion [3, 8]. This process is thought to be phylogenetically older than the cognitive components of empathy (see [7]) and to rely on cortical regions such as the anterior cingulate cortex, the insula [11–14] and the inferior frontal gyrus [15, 16]. On the other hand, the cognitive component of empathy is conceptualized as the capacity to consciously adopt the perspective of others and requires perspective taking as well as mentalizing [3, 17]. The cognitive component of empathy has been shown to rely on the ventromedial prefrontal cortex [16], the superior temporal cortex, precuneous and the temporo-parietal junction [14].

The cognitive and affective components of empathy are often assessed using self-reported measures such as the Interpersonal Reactivity Index (IRI) [18]. The IRI is one of the most commonly used measure of self-reported empathy and decomposes empathy into four constructs [18]. The affective component is measured by the Empathic Concern (i.e., the ‘other-oriented’ feelings of sympathy and concerned for others) and the Personal Distress (i.e., the ‘self-oriented’ feeling of anxiety and discomfort in interpersonal settings) subscales, while the cognitive component is measured by the Perspective Taking (i.e., the spontaneous tendency to adopt the psychological perspective of others) and the Fantasy (i.e., the tendency to identify imaginatively with fictitious characters) subscales.

Pioneer neurogenetic studies have investigated the source of the genetic variability of empathy by examining the effect of specific genetic variants on self-reported empathy [19] and intermediate processes in the brain, such as emotion perception [20]. So far, the oxytocin receptor (*OXTR*) gene is probably the most studied gene with respect to social cognition and social behaviors. For instance, single-nucleotide polymorphisms (SNP) of the *OXTR* genes have been associated with social cognition in adults [19, 21], children [22] as well as in schizophrenic individuals [23]. Amongst these findings, only two SNPs of the *OXTR* gene, rs53576 and rs2254298, have been associated both with self-reported empathy and functional neuroimaging tasks of emotion perception [20, 24]. Accordingly, Rodrigues and colleagues (2009) were the first to report an association between a genetic variant and self-reported empathy by showing that A allele carriers of the rs53576 polymorphism reported less dispositional empathy on the IRI, as well as less accurate judgement of facial expressions. Furthermore, Tost and colleagues (2010) showed that A allele carriers of the rs53576 SNP presented decreased amygdala reactivity during the observation of angry and fearful faces, as well as a greater functional coupling between the amygdala and the hypothalamus during this task. Similarly, A allele carriers of the rs2254298 were also shown to present less self-reported empathy on the total IRI score [21], smaller amygdala volumes [25], as well as “deficient deactivation” of the dorsal anterior cingulate cortex during emotion perception [24]. This convergence of evidences strongly suggests a role of both polymorphisms in the neurobiology of social perception as well as in self-reported empathy. Interestingly, both SNPs are located in the third intron, a non-coding region of the gene, and their functional effects still remain unknown.

To further investigate the genetic factors affecting empathy, a likely genetic candidate is the Brain-derived neurotrophic factor (*BDNF*) Val66Met polymorphism (196 A/G; rs6265), a genetic variant extensively studied for its role in intermediate phenotypes of social perception as well as in psychopathologies associated with social cognitive deficits such as schizophrenia (see [26]). Notably, using similar experimental tasks of social perception as the ones used to study the *OXTR* SNPs, the *BDNF* Val66Met variant has been associated with amygdala reactivity to emotional stimuli in healthy [27] and anxious individuals (Lau et al., 2010). More precisely, Met allele carriers (ValMet / MetMet) were reported to present more reactivity than Val/Val participants to emotional stimuli. Furthermore, Met allele carriers have also been shown to present greater attention biases to social threats, as well as increased connectivity between the amygdala and the ventromedial prefrontal cortex [28]. This polymorphism has been linked to three effects in BDNF signalling: (1) a decreased concentration of pro-BDNF in dendrites (2) a decreased level of pro-BDNF in the secretory granules and (3) a decreased secretion of the molecule in the synaptic cleft [29–31].

Given the crucial role of the amygdala nuclei and the ventromedial prefrontal cortex in social perception and empathy, it is possible that the *BDNF* Val66Met might also play an important role in self-reported empathy. Therefore, we hypothesized that this increased reactivity of social perceptive processes in Met allele carriers might lead to altered self-reported empathy.

To test this hypothesis, we investigated the effect of the *BDNF* Val66Met polymorphism on one of the most commonly used self-reported measure of empathy, the IRI [18]. Furthermore, we tested whether this SNP could have a distinct influence on self-reported empathy beyond the effect of the two SNPs of the *OXTR* gene previously associated with the IRI [19, 21] and to intermediate phenotypes of emotion perception [20, 24]. Also, we investigated the interaction effect of rs53576 and rs2254298 on self-reported empathy as the influence of these polymorphisms have until now only been considered separately. We hypothesized that Met participants of the *BDNF* Val66Met polymorphism would report less self-reported empathy than Val/Val participants and that the A allele of both *OXTR* SNPs would be associated with less self-reported empathy.

## Materials and Methods

### Participants and Procedure

113 young adults (57 females; mean age = 24.14; SD = 4.44) were recruited using the University Laval electronic newsletter. The sample size was determined using effect sizes from previous studies that documented the effect of the rs53576 and the rs2254298 on self-reported empathy. Cohen’s d were computed and averaged from all published articles that reported a significant effect of either SNPs on self-reported empathy [19, 21, 23, 32, 33]. One article was excluded from the analysis since insufficient information was reported [33]. The result indicated an average Cohen’s d of 0.6, which is considered to be a medium size effect according to Cohen’s guidelines [34]. A prospective Power analysis achieved using G*Power 3 [35] indicated that a sample size of 110 participants was sufficient to obtain a power of .7 with our statistical model (i.e., MANOVA). The power level was set at .7, which represents a 30% risk of not detecting an effect of genotypes (i.e., making a type II error). Participants were right-handed, self-reported no diagnosis of psychopathologies or neurological conditions and reported not taking any prescription medication. Only self-reported Caucasians of European ancestry were included to avoid population stratification artefacts, as this is the most accessible population in Quebec City. Inform consent was obtained in writing. Three participants were excluded from further analyses because they provided too little saliva for genetic analyses. The study was approved by the local ethics committee (i.e., Institut de réadaptation en déficience physique de Québec).

### Genotyping procedure

Saliva samples were collected using the Oragene DNA self-collection kit (DNAGenotek). DNA was extracted using the Blood & cell culture DNA maxi kit (Qiagen) and concentration evaluated by fluorescence (Qubit). Some 50 nanograms of DNA was used to amplify the region of the *BDNF* Val66Met polymorphism using a real-time polymerase chained reaction cycler (Ligthcycler 480, Roche) and a TaqMan 5’ nuclease assay kit (Life Technologies). PCR was made using 0.5 ul of 10X PCR MasterMix (Roche), 0.25 ul 40X TaqMan assay, 50 ng DNA in a final volume of 5 uL. PCR run included a denaturing step of 10 min-95°C followed by 35 PCR cycles (1 min 95°C, 1 min 55°C, 1 min 72°C).

### Empathy Questionnaire

The Interpersonal Reactivity Index (IRI; [18]) is a 28-item self-reported scale assessing empathy comprising four subscales: Perspective Taking, Empathic Concern, Fantasy and Personal Distress. Each subscale includes 7 items rated on a 5-point Likert scales (1 = Does not describe me well; 5 = Describes me well) and were scored using criteria developed by Davis [18]. The test re-test validity of the IRI subscales has been reported to range from .63 to .71 [36]. In our sample, all subscales presented acceptable internal consistency (Cronbach’s alpha: Perspective Taking = .70; Fantasy = .82; Empathic concern = .69; Personal Distress = .76). Data were analyzed using IBM SPSS Statistics version: 20.

### Data Analysis

To determine the effect of *BDNF* Val66Met on self-reported empathy, a MANOVA was conducted on the four IRI subscales as dependant variables with the *BDNF* Val66Met, the *OXTR* rs53576 and the *OXTR* rs2254298 as independent variables, as well as the interaction between the two *OXTR* SNPs. Significant main effects and interactions were further decomposed using separate stepwise discriminant analyses to determine individual contribution of subscales to discriminate genotypes (see [37]). Each discriminant analysis was conducted by considering as covariates the genotypes present in the MANOVA but not considered in this particular discriminant analysis. For instance, to determine the contribution of each IRI subscale to discriminate the *BDNF* genotype, individual scores on the IRI subscales were adjusted for the effect of the two *OXTR* SNPs and their interaction. Adjusted IRI scores were obtained by taking the non-standardised residuals of the regression between each IRI subscales and covariates (genotypes), which is similar to the procedure used to control for covariates in analyses of covariance [38, 39]. To evaluate the interaction between the *OXTR* polymorphisms, we determined if the adjusted IRI subscales could discriminate the four groups created by the interaction of *OXTR* polymorphisms in the discriminant analysis.

## Results

The genotype distribution of the Val66Met, rs53576 and rs2254298 are presented in Table 1. None of the distribution differed significantly from the expected numbers calculated on the basis of observed allele frequencies according to the Hardy–Weinberg equilibrium (*p* > 0.05). Participants in the Met and Val/Val groups did not differ on age (*t* (108) = -.679; *p* = .50) and males and females were equally distributed between groups (χ2 (1, *N* = 110) = 3.379; *p* = .07) (see Table 2). Furthermore, on both *OXTR* SNPs, participants in the AA/AG and the GG groups did not differ on age (rs53576: *t* (108) = 1.68; *p* = .10; rs2254298: *t* (108) = -.729; *p* = .47) and the sex of participants was equally distributed between genetic groups (rs53576: *χ2* (1, *N* = 110) = 0.324; *p* = .56; rs2254298: *χ2* (1, *N* = 110) = 1.01; *p* = .32) (see Table 2). For each polymorphism, as achieved in most previous studies, homozygous carriers of the mutant alleles were pooled with heterozygous carriers to allow sufficient statistical power for comparisons. The BDNF genotype presented no association with either the rs53576 (*ϕ* = .062 *p* = .52) or the rs2254298 (*ϕ* = .069 *p* = .47) polymorphisms, but the two OXTRs SNPs presented a weak negative association (*ϕ* = -.267 *p* = .005), which represent a certain linkage disequilibrium These results indicates that participants of one of the two groups on the first SNP were more likely to be in the other group on the second SNP. This linkage disequilibrium is expected since both SNPs are located in the same intron and should not influence the results of the MANOVA for two reasons: first, the postulates of MANOVA allow independent variables to be correlated and second, the association between two independent variables do not indicate that they will interact in their effect on another dependant variable.

**Table 1:** Distribution of genotypes.

SNPs	Genotypes	n	Total	p-EHW
*OXTR* rs53576	GG—GA—AA	54/45/11	110	.72
*OXTR* rs2254298	GG—GA—AA	84/24/2	110	.85
*BDNF* Val66Met	Val/Val—Val/Met—Met/Met	70/34/6	110	.49

SNP = Single nucleotide polymorphism; p-EHW = p values of the Hardy-Weinberg equilibrium.

**Table 2:** Demographics of participants as a function of the Val66Met polymorphism, rs53576 and rs2254298 polymorphisms.

	**Val66Met *BDNF***						
	**ValVal**			**ValMet / MetMet**			
**Demographics**	**N**	**male**	**female**	**N**	**male**	**female**	**p**†
Sex	70	55.7%	44.3%	40	37,5%	62,5%	.07
	**N**	**mean**	**S.E.M.**	**N**	**mean**	**S.E.M.**	**P**^‡^
Age	70	24.36	.62	40	23.75	.48	.50
	***OXTR* rs53576**						
	**GG**			**AA/AG**			
**Demographics**	**N**	**male**	**female**	**N**	**male**	**female**	**p**†
Sex	54	51.9%	48.1%	56	46.4%	53.6%	.56
	**N**	**mean**	**S.E.M.**	**N**	**mean**	**S.E.M.**	**P**^‡^
Age	54	23.41	0.64	56	24.83	0.56	.10
	***OXTR* rs2254298**						
	**GG**			**AA/AG**			
**Demographics**	**N**	**male**	**female**	**N**	**male**	**female**	**p**†
Sex	84	46,4%	53.6%	26	57.7%	42.3%	.32
	**N**	**mean**	**S.E.M.**	**N**	**mean**	**S.E.M.**	**P**^‡^
Age	84	24.31	0.51	26	23.58	0.77	.47

n = number of available data points, S.E.M. = standard error of the mean,

^†^ = Chi-square test,

^‡^ = t-tests.

The MANOVA conducted on the four IRI subscales indicated significant main effects of the *BDNF* Val66Met (*F* (4, 102) = 2,69; *p* = .035; partial η^2^ = .10; Observed power = .73), rs53576 (*F* (4, 102) = 3,59; *p* = .009; partial η^2^ = .12; Observed power = .86), rs2254298 (*F* (4, 102) = 2,66; *p* = .037; partial η^2^ = .09; Observed power = .72), as well as an interaction between the *OXTR* SNPs (*F* (4, 102) = 2,53; *p* = .045; partial η^2^ = .09; Observed power = .70) (Table 3). These results indicate that the linear combination of the IRI subscales is associated with the *BDNF* Val66Met polymorphism and to the interaction of the *OXTR* SNPs. An exploratory full factorial MANOVA was also conducted and revealed no three-ways interaction (*F* (4, 99) = 0,57; *p* = .731; partial η^2^ = .02; Observed power = .17), nor two-ways interactions between the BDNF Val66Met and either OXTR SNPs (rs53576: *F* (4, 99) = 0,20; *p* = .94; partial η^2^ = .008; Observed power = .09; rs53576: *F* (4, 99) = 0,42; *p* = .79; partial η^2^ = .017; Observed power = .15). The low power of this analysis, however, warrants caution in the interpretation of these results.

**Table 3:** Main effects and interactions of the *BDNF* Val66Met, *OXTR* rs52576 and rs2254298 polymorphisms on the linear combinations of the 4 subscales of the IRI.

	MANOVA						
	Wilk’s Λ	F	df	error df	p	partial η^2^	Observed power
*BDNF* Val66Met	.91	2.69	4	102	.035*	.10	.73
*OXTR* rs53576	.88	3.58	4	102	.009*	.12	.86
*OXTR* rs2254298	.90	2.69	4	102	.037*	.09	.72
rs53576 * rs2254298	.91	2.53	4	102	.045*	.09	.70

* *P* < .05.

To further study how individual IRI subscales are associated with the *BDNF* Val66Met and to the interaction of the *OXTR* SNPs, we introduced the IRI subscales as predictors in two separate stepwise discriminant analysis to discriminate (1) *BDNF* Val66Met polymorphism and (2) the four groups created by the interaction of the *OXTR* SNPs (see Procedure). The results indicated that a significant function discriminates the *BDNF* polymorphisms using the Perspective Taking, Fantasy and Empathic Concern subscales when the effect of the SNPs of the *OXTR* on individual subscales are considered (Λ = .910, χ2 (3) = 10.07, *p* = .018; Canonical R^2^ = .09). The correlations between the subscales and the discriminant function indicate that the Perspective Taking (*r* = -.58) and the Fantasy subscale (*r* = .42) make significant unique contribution to the prediction of the *BDNF* Val66Met genotype while the Empathic Concern (*r* = .28) and the Personal Distress (*r* = .19) subscales do not present a significant association with this prediction. Specifically, the Met group membership is predicted by less Perspective Taking and more Fantasy. Furthermore, the results of the second stepwise discriminant analysis indicate that only Personal Distress is used to discriminate the *OXTR* groups created by the interaction of the rs53576 and the rs2254298 polymorphism when the effect of the SNP of the *BDNF* on individual subscales is considered (*Λ* = .909, *χ2* (3) = 10.21, *p* = .017; Canonical R^2^ = .09). More precisely, Personal distress significantly discriminates between individuals presenting A alleles on both SNPs from individuals of the three other genotypes. A lower score on Personal Distress predicts membership to the group of participants presenting A alleles on both SNPs of the *OXTR*.

## Discussion

Our results provide the first evidence of an association between the *BDNF* Val66Met polymorphism and self-reported empathy. Specifically, the Met group membership is predicted by less Perspective Taking and more Fantasy when the effects of the targeted *OXTR* SNPs are considered. Furthermore, our results indicate that the rs53576 and rs2254298 SNPs of the *OXTR* may interact to confer their effect on self-reported empathy. More precisely, our results indicate that less Personal Distress is associated with participants presenting the A alleles on both *OXTR* SNPs when the effect of the *BDNF* Val66Met is considered. By uncovering dissociable effects of the *BDNF* Val66Met and the *OXTR* SNPs, our results make a significant contribution to our understanding of the genetic factors affecting self-reported empathy.

The fact that less Perspective Taking and more Fantasy predict the Met group membership indicates a differential effect of the *BDNF* Val66Met on the two subscales. In fact, even though the two subscales are often regrouped under the construct of Cognitive empathy, they describe types of social interactions that differ in terms of the level of personal involvement they require. On the one hand, the Fantasy construct mainly represents the tendency to identify with fiction characters, which does not necessitate personal involvement. On the other hand, Perspective Taking conceptualizes the cognitive aspects of empathy in the context of actual social interactions requiring a high level of personal involvement. There are indeed evidences that *offline* (i.e., inferential representation of a social situation requiring no direct involvement) and *online* (i.e., direct involvement in a social situation) social cognitive processes recruit distinct regions of the ventromedian prefrontal cortex [40]. In fact, several results support the hypothesis that different social cognitive processes are recruited in situations where individuals are directly in interaction with others and when they are simply observing others (for a review, see [41]). One possibility is that the ValVal genotype may favour the understanding of others in the context of direct social interactions, which requires personal involvement, as opposed to the ValMet / MetMet genotypes that may favour the capacity to adopt the point of view of fiction characters in situations that do not require personal involvement.

Interestingly, the biological functionality of the BDNF Val66Met can provide hypotheses regarding the discrepancy of its effect on the Perspective Taking and the Fantasy subscales. The BDNF Val66Met has indeed been reported to influence experience-dependant plasticity [29–31], a process that might affect differently the *online* and *offline* processing of social information. For instance, Perspective Taking during *online* social interactions might require rapid plasticity to coordinate social actions and to facilitate the integration of multiple social cues while *offline* social processing might not rely as heavily on this plasticity. For instance, *offline* social processing such as reading a book, daydreaming and fantasizing do not require the coordination of social responses, and the relevant information can often be integrated much more slowly then typical *online* social interactions. It is even possible to imagine that too much plasticity might generate interference in situations where less plasticity would be required. Future studies could test these hypotheses by investigating the propensity for the cognitive component of empathy in *online* and *offline* social interactions in order to further determine the role of the *BDNF* Val66Met polymorphism in these social cognitive processes.

Furthermore, if *online* and *offline* social interactions present distinct neural and cognitive processes, do they also present distinct levels of heritability? In fact, some evidences indicate that genetic variants might be responsible for 34% to 47% of the variance of empathy [6], but little is known about the heritability of its individual components. If *online* and *offline* social interactions indeed serve different purposes with different evolutionary advantages like some authors have proposed [41], it is possible that they may also be influenced by different genetic factors and have different levels of heritability. For instance, reflecting on social interactions from an observer’s point of view (*offline* social interactions) might allow to elaborate and fine-tune general rules of social interactions in order to guide future behaviour which cannot be easily achieved during *online* social interactions that are emotionally involving and that might have direct consequences for the individual’s survival. Interestingly, our results do not seem to support this hypothesis since we observed that one genetic factor, the BDNF Val66Met, accounted for part of the variability of both these constructs. As our results do not allow the determination of the heritability of these components of empathy, future studies are needed to investigate this specifically.

Our results also build on the existing literature linking the *OXTR* rs53576 and the *OXTR* rs2254298 to self-reported empathy and highlight the necessity for a multivariate approach. It is in fact possible that genetic variants such as the rs53576 [20] and the rs2254298 [24] acting on the neurobiological level, may have to simultaneously interfere with the perception of emotional stimuli to affect Personal Distress, an higher-order cognitive construct related to the reactivity to emotion perception. Therefore, using multivariate approaches will most likely further improve our capacity to determine the many genetic factors contributing to self-reported empathy and more importantly these approaches could also help bridge the gap between intermediate neural phenotypes and behavioural measurements of psychopathologies.

The fact that *BDNF* Val66Met is associated with empathy also opens new research avenues for the study of psychopathologies involving social-cognitive deficits, such as autism or schizophrenia. For instance, schizophrenia has been associated both with decreased self-reported Perspective Taking [42] and with the *BDNF* Val66Met polymorphism (see [26]). Our results provide new evidences suggesting a link between Perspective Taking and the *BDNF* Val66Met polymorphism, which may partly explain why schizophrenia was associated independently with Perspective Taking and the *BDNF* Val66Met polymorphism. Interestingly, the rs2254298 polymorphism has already been associated with the affective component of empathy in schizophrenic patients (see [23]) and studying the role of the *BDNF* Val66Met on Perspective Taking could further improve our understanding of the genetic factors contributing to social cognitive difficulties in this psychopathology.

One must although keep in mind that these results should be replicated in independent studies to further establish their validity. While our approach aimed at controlling for confounding genetic factors, replication remains one of the most effective ways to establish the validity of these results, since every study that use random sampling of the population can unknowingly report false positive.

In conclusion, this study provides results indicating for the first time a link between the *BDNF* Val66Met polymorphism and self-reported empathy that goes beyond the previously reported effects of the rs53576 and rs2254298 SNPs. The results also further builds on the previous literature suggesting a link between the rs53576, the rs2254298 and self-reported empathy and highlights the importance of studying gene X gene interactions to better understand the genetic factors contributing to interindividual variability in self-reported empathy.

## Supporting Information

S1 Data Data underlying the findings reported in the manuscript. (XLSX)
